# Calculation of Costs of Pregnancy- and Puerperium-related Care: Experience from a Hospital in a Low-income Country

**DOI:** 10.3329/jhpn.v28i3.5555

**Published:** 2010-06

**Authors:** M.G. Sarowar, E. Medin, R. Gazi, T.P. Koehlmoos, C. Rehnberg, R. Saifi, A. Bhuiya, J. Khan

**Affiliations:** ^1^ Health Economics Unit, Department of Learning, Informatics, Management and Ethics, Karolinska Institute, Stockholm, Sweden; ^2^ Health System and Economics Unit, ICDDR,B, GPO Box 128, Dhaka 1000, Bangladesh; ^3^ Faculty of Economics and Administration, University of Malaya, 50603 Kuala Lumpur, Malaysia

**Keywords:** Cost calculation, Costs and cost analysis, Health expenditure, Healthcare cost, Hospital cost, Maternal health, Micro-costing, Bangladesh

## Abstract

Calculation of costs of different medical and surgical services has numerous uses, which include monitoring the performance of service-delivery, setting the efficiency target, benchmarking of services across all sectors, considering investment decisions, commissioning to meet health needs, and negotiating revised levels of funding. The role of private-sector healthcare facilities has been increasing rapidly over the last decade. Despite the overall improvement in the public and private healthcare sectors in Bangladesh, lack of price benchmarking leads to patients facing unexplained price discrimination when receiving healthcare services. The aim of the study was to calculate the hospital-care cost of disease-specific cases, specifically pregnancy- and puerperium-related cases, and to indentify the practical challenges of conducting costing studies in the hospital setting in Bangladesh. A combination of micro-costing and step-down cost allocation was used for collecting information on the cost items and, ultimately, for calculating the unit cost for each diagnostic case. Data were collected from the hospital records of 162 patients having 11 different clinical diagnoses. Caesarean section due to maternal and foetal complications was the most expensive type of case whereas the length of stay due to complications was the major driver of cost. Some constraints in keeping hospital medical records and accounting practices were observed. Despite these constraints, the findings of the study indicate that it is feasible to carry out a large-scale study to further explore the costs of different hospital-care services.

## INTRODUCTION

The financing of health systems are a key determinant of health and well-being of population (1). Due to poor allocation of resources within the public sector in many low-income countries, healthcare financing heavily relies on out-of-pocket expenditure by patients. At the same time, healthcare expenditure is increasing in most countries across the world. This increased healthcare expenditure and the heavy out-of-pocket payment limit people in seeking healthcare or in continuing their treatment. In many low-income countries, poor people often fail to avail of necessary healthcare services due to financial constraints (2) and often face catastrophic financial burden to meet their healthcare expenditure (3, 4). Universal coverage through sustainable methods of health system financing is a core interest of many health research organizations, including the World Health Organization (5).

Costing is a major activity to define future service packages, plan payment methods, and measure the efficiency of the healthcare system (6). Other than designing benefit packages and planning payment methods, calculation of costs of different medical and surgical services has numerous uses that include monitoring the performance of service-delivery, setting the efficiency target, benchmarking of services across all sectors, considering investment decisions, commissioning to meet health needs, and negotiating revised levels of funding (7). Barnum and Kutzin detailed the necessities of knowledge about unit costs of different healthcare services for planning recurrent budgets as an indicator of efficiency and to inform pricing of services to patients and relevant policy-makers (8). Such information can be used for suggesting the means of increasing the hospital efficiency through adjustments to factor-mix, changes in the length of stay, and an improved use of the referral system and pricing information for the consumer (8).

### Background

Most healthcare systems involve a mixture of public and private provisions of healthcare (9). In most low-income countries, the majority of people receive most of their healthcare from the private sector as in South Asia where three-quarters (75%) of health-service provision take place outside the public sector (10, 11). Bangladesh also has a thriving private sector (12). There are 3,976 healthcare facilities in the public sector and 975 privately-run hospitals or clinics in Bangladesh (13). Bangladesh National Health Accounts 1999–2001 stated, “In response to the growing disappointment in the role of public health sector, the number of private-run facilities has increased. An estimated 15% growth has been observed between 1996 and 2000 in this sector” (13).

Information on the cost of medical care is fundamental for developing an equitable system which can increase access to healthcare services through standardization of prices and increasing efficiency of the hospitals. A treatment-specific calculation of costs could be useful for initiating a policy discussion. Cost-related information, specific to treatments or procedures, is necessary for benchmarking the prices for different medical and surgical care services, which can even be used for developing suitable payment methods, such as fee for service, case-based payment, or diagnostics-related groups (DRGs), etc. to healthcare providers.

In absence of a pricing policy in the private sector and because of case-based payment systems in the public sector, no price-benchmarking has been established in Bangladesh. Patients with the same disease pay different fees for the same services at the same facilities (14). There are several dimensions that can be used for explaining the variation in fees in the private market; for example, fees may vary according to the social status of patients. Three high-status occupational groups, such as doctors, senior government officials, and big businessmen, have the highest probability of receiving the unofficially-discounted price (14).

As part of two prospective research projects on calculation of costs of maternal and child-healthcare services in Bangladesh, a case study was conducted at a secondary care hospital in its obstetric care unit treating conditions relating to pregnancy and puerperium, a six-week period following the birth.

The aim of this case study was to calculate the cost of 11 different pregnancy- and puerperium-related cases admitted to a secondary care hospital and to identify the practical challenges of carrying out this type of cost-calculation study in Bangladesh.

## MATERIALS AND METHODS

### Methods

The study used a mixed method of ‘micro-costing’ and ‘step-down cost allocation’ for collecting information on different cost items. The information was used for calculating the final unit cost for each service or procedure.

Micro-costing is a meticulous, bottom-up time- and money-consuming method whereas step-down allocation is a macro, top-down approach that is easy to implement but not considered highly reliable and accurate (15). The literature on costing recommends the adoption of a mixed method in bottom-up and top-down approach when a sophisticated cost-accounting system is not present. The mixed method is also suitable to capture detailed information on recurrent cost items, such as medicines, diagnostic tests, blood transfusion, foods, fees, etc., and on hospital-shared overheads and capital cost items. The present study employed micro-costing for the recurrent cost items and step-down cost allocation for the hospital-shared overheads and capital items. Zsolt and Smith discussed in details a suitable method of costing in different settings and reality (15).

### Source of data

The study was conducted at the inpatient obstetric and labour ward of General Hospital run by public financing in Dhaka, the capital city of Bangladesh. This hospital has facilities for secondary-level care for most general diseases and surgical conditions, with both outpatient and inpatient departments. A structured form was developed and pre-tested so that the cost data from the medical records of patients could be uniformly extracted. The hospital authority provided medical records for the July 2006–June 2007 period. Cost items were classified as direct, indirect (or intermediate), and capital cost items (16). Costs relating to the treatment procedures of the patients were classified as direct while cost expenses not relating to the treatment plan were considered indirect cost. Only buildings and beds were considered capital goods. In Bangladesh, the public hospitals are run by government financing and some occasional voluntary national and international donations (mostly goods). Although hospitals are supposed to bear all the expenses relating to the treatment and diagnostic procedures in the inpatient wards, the reality is different from expectation. The treatment-related costs are shared by both hospital (through government funding) and patient through out-of-pocket expenditure.

### Measuring costs

#### Direct costs

Most direct costs, such as costs of medicines, diagnostic tests, blood transfusions, etc., were retrieved from the medical records of the patients. Medicines supplied from the hospital were accounted according to the price list of purchase by the hospital, and the market price was used for accounting for medicines bought by out-of-pocket expenditure. Cost of food was assumed to be fixed at an average rate (US$ 0.65 per day) for all patients supplied by the hospital. There were two fixed rates for operation theatre (OT) costs: the cost was US$ 3.5 per patient for a minor surgery, and US$ 7.5 per patient for a major surgery. These rates were applied, wherever applicable, as OT cost for each patient.

#### Indirect costs

We identified numerous indirect costs, including linens and laundry, cleaning and nightguards, utilities, electricity, gas, telephone, and water. The entire utility bill of one year was collected from the hospital accounting office. As a portion of costs is shared by several departments in the hospital, we used a shadow costing for distributing the total indirect costs to the specific wards. As the total number of patients in the hospital was unavailable, we could not use the share of patients in the study ward in relation to the total number of patients in the hospital for calculating the shared overhead costs. Therefore, we used the proportion of the floor-space of the study ward in relation to the whole hospital for assigning the shared costs. The study ward occupies 3% of the total floor-space, and we, consequently, have taken to be considered 3% of the overhead costs.

#### Capital costs

Costs of assets are usually invested in a bulk amount and used over time (16). The major capital cost items were identified as buildings, beds, and vehicles. The ambulance was excluded from analysis as the ambulance was not used for the hospital. We measured the total floor-space of the ward and valued it according to the government rate of per square feet floor-space. The cost of the floor-space of the study ward was annualized to calculate the annual price of the floor-space for one year. There was no valuation record of beds and other accessories in the ward. We took the market price and annualized the price of building (floor-space) and the beds in the ward. For annualization, we have considered 30 years as the functional life-years of the buildings and discounted at the rate of 5%.

#### Calculation of unit costs

We adopted a mixed method—a combination of micro-costing and a modified form of step-down cost accounting model for the calculation of unit costs. We defined the obstetric and labour ward as the final cost centre and assigned different costs as line items under this cost centre. The total cost for every cost item was divided by the number of patient-bed days to get the cost of each item per patient per-bed day. We then added all the cost items per patient bed-day and multiplied by the length of stay of each individual patient to get the total cost for each patient.

## RESULTS

There was no definite method of labelling the diagnoses in the medical records of the patients. We have taken the diagnoses as these were written in the clinical records of the patients. Eleven kinds of diagnosis were found from the medical records. Data were collected from the medical records of 162 patients. The cases were diagnosed as: pregnancy with hyper-emesis; pregnancy with urinary tract infection (UTI); pregnancy with respiratory tract infection (RTI); full-term pregnancy with foetal complication; full-term pregnancy with maternal complication; full-term pregnancy with labour pain; pregnancy with generalized weakness; abortion and related cases; infertility and others; pregnancy with fever; and one case of ectopic pregnancy. In total, 146 patients completed the treatment procedure, and 16 had taken an early discharge without completing the treatment. The mean age of the patients was 23 (range 16–45) years. We included all the patients in the analysis, including those who did not complete treatment. All costs were estimated primarily in Bangladeshi taka and later converted to US dollar at the mean exchange rate prevalent in December 2006 (US$ 1=Tk 69.49) (http://www.bangladesh-bank.org).

Of the 162 patients, 108 were managed with medical treatment while the remaining 54 had surgical treatment. Forty patients were labelled ‘complicated’ because of clinical consequences and length of stay (more than 10 days). Complication status was specified only in a few original medical records, and most of them were post-operative cases. More than 10 days’ stay in the hospital was taken as a cut-off point by the authors, with a verbal discussion with the hospital doctors from their usual experience. In that hospital's experience, pregnancy with hyper-emesis was mostly a same-day case staying 1–2 days, a caesarean section patient generally required 5–7 post-operative days, and a dilatation and curettage (D&C) case was also a same-day surgical procedure in normal practice.

The length of stay of any patient was dependent on the clinical status and the outcome of the treatment. In this study, the mean hospital stay for all patients was 8.25 (range 1–32) days. Younger mothers or first-time pregnant women were more likely to experience maternal and/or foetal complication and consequently incurred higher treatment costs.

### Calculation of costs

Including all clinical diagnoses, the overall total cost for each patient was US$ 73.45. This cost was contributed by direct costs (US$ 61.43), indirect costs (US$ 8.33), and capital costs (US$ 3.69). Of this overall total cost, the hospital provided US$ 65.70, and the patient's out-of-pocket mean contribution was US$ 7.75 (Table 1).

**Table 1.** Mean cost (US$) for treating one patient at the obstetric ward without considering specific diagnosis

Specific diagnosis	Obstetrics	Mean	Standard deviation
Direct medical cost	162	61.43	34.37 (7.82–205.14)
Indirect cost	162	8.33	4.94 (1.01–32.29)
Capital cost/patient	162	3.69	2.19 (0.45–14.31)
Out-of-pocket	162	7.75	9.71 (0.22–57.92)
Hospital's cost	162	65.70	37.21 (9.06–238.51)
Total cost	162	73.45	41.18 (9.28–251.74)

Figures in parentheses indicate minimum cost and maximum cost

We further calculated the cost for each type of clinical case (Table 2). The mean cost of full-term pregnancy with maternal complication was US$ 93.19, and with foetal complication, it was US$ 93.71. The mean cost of the abortion-related cases was US$ 82.84, in which the high cost mainly was due to the higher number of complicated cases (4 of 5). For the full-term pregnancies with maternal and foetal complications, the longer duration of stay was the main driver of cost.

**Table 2.** Number of cases and costs (US$) of different diagnostic categories

Clinical diagnosis	Frequency	%	Mean cost	Standard deviation
Early pregnancy with hyper-emesis	61	37.65	64.00	40.00 (9.28–218.57)
Early pregnancy with UTI	17	10.49	49.02	23.97 (16.43–106.35)
Early pregnancy with RTI	4	2.47	92.58	30.00 (51.61–123.12)
Full-term pregnancy with foetal complication	23	14.20	93.71	27.04 (62.64–196.98)
Full-term pregnancy with maternal complication	21	12.96	93.19	43.54 (30.69–212.55)
Full-term pregnancy with labour pain	8	4.94	63.47	32.16 (19.15–106.90)
Pregnancy with generalized weakness	12	7.41	66.58	40.27 (17.14–163.03)
Abortion and related diseases	5	3.09	82.84	49.94 (19.22–137.50)
Infertility and others	5	3.09	97.23	91.75 (23.81–251.74)
Pregnancy with fever	5	3.09	82.40	25.41 (57.12–109.14)
Ectopic pregnancy	1	0.62	60.71	-(60.71–60.71)

RTI=Respiratory tract infection;

UTI=Urinary tract infection;

Figures in parentheses indicate minimum cost and maximum cost

In most cases, complications increased the duration of stay almost 2–3 times (Table 3). The mean duration of stay of medically-treated uncomplicated patients was 6.42 days whereas a complicated case had an average duration of stay of 14.47 days. An uncomplicated normal delivery had a duration of stay of 4.88 days while the mean duration of stay for a complicated delivery was 13.67 days.

**Table 3.** Variation in duration (days) of stay and costs (US$) due to complications

Management/procedure	No. of cases (complication)		Mean duration of stay (complication)		Mean cost (complication)	
	Yes	No	Yes	No	Yes	No
Medical treatment	15	93	14.47	6.42	114.19	53.62
Caesarean section	17	19	11.29	8.32	117.12	89.98
Normal delivery	3	8	13.67	4.88	112.46	43.63
Dilatation and curettage of uterus	4	1	17.5	4	147.66	42.88
Surgical toileting	1	1	13	7	115.89	63.52

In obstetric and gynaecological practice, the procedure of D&C is considered a same-day surgery if the case is uncomplicated. However, in this study, the mean duration of stay for an uncomplicated D&C was four days while a complicated D&C involved an average duration of stay of 17.5 days. Costs for D&C increased because of the longer duration of stay due to a complication.

The average cost of a medically-managed uncomplicated patient was US$ 53.62 whereas a complicated case with the same diagnosis incurred almost twice the cost (US$ 114.19). The average cost for performing a D&C for an uncomplicated case was US$ 42.88, and the mean cost of complicated cases was US$ 147.66. The cost for surgical toileting was US$ 63.52, and it was US$ 115.89 for non-complicated and complicated cases (Table 3).

Our study found that the direct cost items were the major contributors to the total unit costs. Within the direct cost items, the salary of the nursing staff (US$ 24.56) was the highest cost item, followed by the physician's salary (US$ 24.25) and out-of-pocket medicine or pharmaceutical costs (US$ 5.21) (Table 4). Among the indirect cost items, cost of food per patient was the highest (US$ 5.35). Food was supplied by external vendors at a fixed price per day per patient. The total cost of food per patient was, thus, directly related to the duration of stay of the patient.

**Table 4.** Mean cost (US$) per patient for each cost item

Type and item cost	Mean cost	Standard deviation
Direct cost		
Medicine (hospital)	2.44	2.96 (0.00–12.49)
Medicine (out-of-pocket)	5.21	6.66 (0.00–49.79)
Physician cost	24.25	14.36 (2.94–93.95)
Nursing staff cost	24.56	14.55 (2.97–95.17)
Investigation cost	2.42	2.25 (0.00–10.39)
Blood transfusion	0.22	1.09 (0.00–5.76)
Indirect cost		
Food	5.35	3.17 (0.65–20.72)
Administration cost	1.10	0.65 (0.13–4.26)
Support services	1.65	0.97 (0.20–6.37)
Utility services	0.24	0.14 (0.03–0.93)
Capital cost		
Cost of floor-space	3.69	2.19 (0.45–14.31)

Figures in parentheses indicate minimum cost and maximum cost

### Constraints and challenges

Two main objectives of this study were to test the methodology of costing studies in a practical setting and to uncover the constraints to implementing these methods in a hospital setting in Bangladesh. In the hospitals of Bangladesh, medical documentation is paper-based, and there is no standardized coding system for labelling clinical diagnoses of patients. All medicines in the prescriptions were written by their local brand names, instead of their generic names; as a result, on occasion, a patient was prescribed one specific brand but was provided a different brand by the hospital or dispensary. Hospital-supplied medicines were generally less expensive than those supplied from other sources. Although most (96.59%) patients received a certain amount of medicines from the hospital supply, the amount was not specified in the medical record or in hospital accounts office. For that reason, to calculate the cost of medicine, total use of medicines had to be isolated from the medical record and multiplied by the unit price taken from the hospital supply list for the study ward.

In the hospitals, the paper-based accounting systems were maintained simultaneously by hospital accountants, store officers, and purchase officers. Some purchase and valuation records were absent, including those for instruments used in the OTs, and for the ambulances and the building. Most instruments in the OT were made available by foreign donors, and as such, no financial documentation was produced within the hospital. The ambulance was also donated and was without financial documentation. For all of these missing costs, except the ambulance, shadow-pricing was conducted by taking the lowest available market price as a proxy. We excluded the ambulance from shadow-pricing as it was not used for the hospital, and no purchase record was available in the office for it.

A few paid staff members received their salaries from the study hospital while working at another hospital, sometimes due to an institutional agreement. In this study, their salaries were calculated in the analysis as they were included in the hospital's budget.

## DISCUSSION

We have calculated the mean hospital cost for 11 specific obstetric diagnostic groups by calculating the overall mean cost for treating one patient in the obstetric ward. It was found that the direct medical cost was the major part of the total cost. Complication was one of the major reasons for the prolonged duration of stay in the hospital, resulting in increased costs. The duration of stay directly correlated with the total cost. Full-term pregnancies with maternal and foetal complications were the two highest cost-incurring clinical cases. Abortion-related cases were the next highest cost-incurring cases. Although as a group, ‘infertility and others’ was actually the most expensive group, we did not include this group in the analysis as this group had five different cases having non-specific diagnoses, and one single case had the highest cost (Tk 17,492=US$ 252.40); the cost was mainly due to the prolonged stay of 32 days for this specific case. This specific case in this group was an outlier; if we exclude this case, the mean cost of the group comes down to US$ 58.74.

There are two studies to get the usual length of stay for normal delivery and caesarean section (14, 18). Amin *et al.* followed 1,212 patients in two private hospitals in Bangladesh and showed that the average length of stay for normal delivery was 2.4 days for a small hospital and 2.6 days for a large hospital, and in the same way, they showed that the average length of stay for caesarean section was 3.5 and 3.7 days for both kinds of facilities (14). Kawnine and colleague also showed in a comparative study by the Health Economics Unit (HEU), Ministry of Health and Family Welfare (MoHFW), Government of Bangladesh, that complications can increase the length of stay (18).

Of the three study hospitals, one non-governmental hospital (LAMB) had 43% of patients with pre-discharge complications that led the average length of stay to be 12 days whereas it was six days in the comparator private hospital. Considering the experience at the study hospital and the two previous studies (14, 18), the 10-day cut-off point was assumed as a maximum rational length to be a non-complicated case; however, some prolonged hospital stay was not consistent with the clinical diagnosis. Of the 40 complicated cases, 15 were managed medically, and the remaining 25 had surgical interventions. Some patients faced complications as an outcome of the surgery. Almost half (n=10) of the full-term pregnancies (n=23) with foetal or maternal complications and the abortion-related cases were labelled ‘complicated’.

As in other low-income countries, healthcare cost-calculation studies are not common in Bangladesh. We found five articles on calculation of costs of maternal health and other hospital-care services in Bangladesh. There is a lack of consistent and acceptable methodologies in these studies. Of these five studies, two are methodologically comparable with our study. The HEU of MoHFW, Government of Bangladesh, conducted both the studies (17, 18).

In Bangladesh, a large-scale study on calculation of hospital costs was carried out by the HEU (MoHFW) and Mymensingh Medical College Hospital (MMCH) for the 1994–1995 financial year (17). Our study has methodological similarities with this study, which used a modified mixed step-down cost accounting method to get an average of cost per patient per day in different wards. They considered eight clinical units (with several wards for each) as final cost-centres and gathered data for direct, indirect and capital costs. As in our study, opportunity costs (work forgone, transportation, waiting-time, family expenses, informal fees, etc.) of different items relating to services were not taken into account in the HEU-MMCH study (17).

Our study is a limited-scale experiment compared to the HEU-MMCH study. There are two main differences between our study and the HEU-MMCH study. First, the HEU-MMCH study placed emphasis on financial auditing aimed at explaining how the budget was used in different sections (surgery, paediatric, etc.) of the hospital whereas our study calculated the cost per patient for each service provided in the hospital. Second, the HEU-MMCH study calculated the cost per patient without specifying any diagnosis whereas we calculated the cost per patient for every diagnostic category. Unlike the HEU-MMCH study, the cost is more disaggregated in the present study, and hence, the outcome information could be more appropriate for developing a fee schedule (reimbursement, capitation, fee-for-service, or price benchmarking).

The HEU of MoHFW carried out another study to calculate total costs (consumers’ and providers’) of a caesarean section. Again, the present study had methodological similarities. The HEU study calculated and compared the costs of a caesarean section among a tertiary-level public hospital, a non-profit private hospital, and a for-profit private hospital, through employing a financial accounting approach. The HEU study considered both recurrent and capital costs relating to patients and service providers and found a wide variation in costs among the three types of providers: cost of a caesarean section was US$ 62.75 at a non-profit private hospital; US$ 124.50 at the tertiary-level public hospital; and US$ 283 at a private for-profit hospital. The private for-profit hospital had almost five times higher cost than the non-profit private hospital and more than two times higher than the public hospital. Based on the findings, this study concluded that the non-profit hospitals can provide the same service at a lower cost.

In the three other studies, the cost or expenditure of different maternal healthcare services in Bangladesh was calculated (19–21) but in a very different way from the objectives and methods of the present study. For example, Nahar and Costello calculated only the patients’ out-of-pocket expenditure of a normal delivery and caesarean section at a secondary hospital in Dhaka (19). This study also accounted further for the patients’ out-of-pocket expenditure by including items, such as travel, medicines, blood transfusion, food, admission fee, and tips.

### Limitation

One limitation of the present study is that it was carried out using only one secondary care-level hospital. The sample is small, which raises questions about generalizability. However, the findings indicate that it is feasible to use this methodology for conducting costing studies in Bangladesh. Although the study does not address efficiency in the delivery of maternal healthcare services, the possibility exists to conduct a cross-study comparison with the previous maternal health-costing studies in Bangladesh, which, to some extent, indicates the efficiency.

### Implications

Disease-specific cost calculated from a representative sample can be a basis for developing a fee schedule for both public and private hospitals in Bangladesh. The implication of the present study is to reduce the information gap about cost of medical care among healthcare leaders, service providers, and consumers.

The results of the study indicate that estimation of costs in a similar setting in Bangladesh is possible. Such information would be helpful for policy-makers in planning a reimbursement system for the public hospitals. By comparing the costs of maternal care in other sectors, e.g. private for-profit and non-profit, the policy-makers can find information on effective resource allocation regarding maternal care services. This type of information can potentially be incorporated for supporting the medical care system with public-private mix in Bangladesh. Further, costing can be used for informing the value of incentivized, government-sponsored financing tools, such as the demand-side finance voucher scheme for maternal health (22, 23).

### Conclusions

The study calculated the mean cost for 11 different admitted groups of subjects relating to pregnancy and puerperium. Complication of disease and the duration of stay were the two major reasons of the total costs**;** as an example, a non-complicated medically-managed patient costs US$ 53 whereas the complicated patient costs US$ 114. Some constraints in keeping of hospital medical records and accounting practices were observed. Examples of these constraints included very non-specific and inconsistent practice of writing diagnoses in the medical record of patients and lack of financial records for donated goods, such as major medical equipment. Despite these constraints, the findings of the study indicate that it is feasible to carry out a large-scale study to further explore the costs of different hospital-care services. The study also illuminates current short-comings in the hospital accounting and administrative practices. Findings from a nationally-representative sample may produce potential evidence to inform policy-makers in planning healthcare financing methods for the public and private healthcare sectors in Bangladesh.

## ACKNOWLEDGEMENTS

The study was funded by the Swedish International Development Cooperation Agency's (Sida's) Department for Research Cooperation (SAREC)
